# Effect of the Addition of Inorganic Fillers on the Properties of Degradable Polymeric Blends for Bone Tissue Engineering

**DOI:** 10.3390/molecules29163826

**Published:** 2024-08-12

**Authors:** Stanisław Marecik, Iwona Pudełko-Prażuch, Mareeswari Balasubramanian, Sundara Moorthi Ganesan, Suvro Chatterjee, Kinga Pielichowska, Ravichandran Kandaswamy, Elżbieta Pamuła

**Affiliations:** 1 Department of Biomaterials and Composites, Faculty of Materials Science and Ceramics, AGH University of Krakow, Al. Mickiewicza 30, 30-059 Krakow, Poland; smarecik@agh.edu.pl (S.M.); ipudelko@agh.edu.pl (I.P.-P.); 2 Department of Rubber and Plastics Technology, Madras Institute of Technology Campus, Anna University, Chromepet, Chennai 600 044, Tamil Nadu, India; venibala18@gmail.com (M.B.); sundaramoorthi1997@gmail.com (S.M.G.); 3 Department of Biotechnology, Golapbag Campus, University of Burdwan, Burdwan 713 104, West Bengal, India; soovro@yahoo.ca

**Keywords:** PLA scaffolds, bone tissue engineering, porous scaffolds, composite scaffolds, polymer blends

## Abstract

Bone tissue exhibits self-healing properties; however, not all defects can be repaired without surgical intervention. Bone tissue engineering offers artificial scaffolds, which can act as a temporary matrix for bone regeneration. The aim of this study was to manufacture scaffolds made of poly(lactic acid), poly(ε-caprolactone), poly(propylene fumarate), and poly(ethylene glycol) modified with bioglass, beta tricalcium phosphate (TCP), and/or wollastonite (W) particles. The scaffolds were fabricated using a gel-casting method and observed with optical and scanning electron microscopes. Attenuated total reflectance-Fourier transform infrared (ATR-FTIR), differential scanning calorimetry (DSC), thermogravimetry (TG), wettability, and degradation tests were conducted. The highest content of TCP without W in the composition caused the highest hydrophilicity (water contact angle of 61.9 ± 6.3°), the fastest degradation rate (7% mass loss within 28 days), moderate ability to precipitate CaP after incubation in PBS, and no cytotoxicity for L929 cells. The highest content of W without TCP caused the highest hydrophobicity (water contact angle of 83.4 ± 1.7°), the lowest thermal stability, slower degradation (3% mass loss within 28 days), and did not evoke CaP precipitation. Moreover, some signs of cytotoxicity on day 1 were observed. The samples with both TCP and W showed moderate properties and the best cytocompatibility on day 4. Interestingly, they were covered with typical cauliflower-like hydroxyapatite deposits after incubation in phosphate-buffered saline (PBS), which might be a sign of their excellent bioactivity.

## 1. Introduction

Bone injuries are often of a critical size for which conventional treatments are not sufficient, thus effective therapy requires a surgical intervention and/or tissue engineering approach [1,2]. The latter needs synthetic substrates that mimic the extracellular matrix of bone and, when combined with different active substances or cells, provide an effective treatment.

Bone tissue engineering (BTE) is based on mimicking bone structure, provided by three-dimensional (3D) scaffolds [3,4]. Scaffolds are structures designed to ensure a supportive framework for cell attachment, proliferation, and differentiation. They can be made from biocompatible materials of natural origin [5] or synthetic polymers [6,7]. The structure of the scaffold should mimic the structure and functions of the extracellular matrix of natural bone, providing the necessary mechanical strength and porosity to facilitate nutrient diffusion and waste removal [8,9]. By combining the structural support of the scaffolds with the regenerative capacity of cells, tissue engineering aims to recreate viable, functional bone tissue that can be used to repair or replace damaged or diseased bone, offering a significant advance over current therapeutic options [10].

Poly(lactic acid) (PLA) demonstrates high biocompatibility, biodegradability [11], and good mechanical properties [12], making it an ideal candidate for bone scaffolds. In addition, PLA can be easily processed using various techniques [13,14,15]; however, it is characterized by relatively high brittleness and slow degradation rates [16]. To overcome these issues, PLA can be blended with poly(ε-caprolactone) (PCL), which is distinguished by greater flexibility and also high biocompatibility [17,18]. Its controlled degradation rate can be adapted to the specific requirements of the application. PCL is also easy to process with various techniques. Nevertheless, PCL has a lower mechanical strength and degrades more slowly than PLA [19]. The blend of PLA and PCL combines the advantages of both materials to produce scaffolds with optimal properties for BTE.

In addition to PLA and PCL, poly(propylene fumarate) (PPF) and poly(ethylene glycol) (PEG) are two other polymers that have gained attention in BTE due to their unique properties [20,21,22,23,24]. PPF is a biodegradable unsaturated polyester that can be cross-linked in situ, providing customizable mechanical properties and degradation rates [25]. PEG is a hydrophilic polymer known for its biocompatibility; although, it has some drawbacks, such as poor mechanical strength and limited cell attachment [26]. However, when incorporated into polymer blends, PEG can act as a plasticizer, improve the hydrophilicity and mechanical properties of the scaffolds, as well as facilitate the controlled release of the embedded bioactive molecules. The combination of PLA, PCL, PPF, and PEG creates a synergistic effect, where each component contributes to the overall performance of the scaffold, making it more suitable for BTE applications.

Even though the polymer blends exhibit good biocompatibility, they can be enriched with ceramic additives, such as beta tricalcium phosphate (TCP), wollastonite (W), or bioglass (BG) to improve their compatibility with bone tissue [27]. TCP, which belongs to a group of bioactive ceramics, promotes mineralization and integration with natural bone tissue, which accelerates its regeneration process [28]. Moreover, TCP is resorbable due to the activity of osteoclasts. It allows the scaffold to gradually degrade, releasing calcium and phosphate ions that contribute to bone mineralization and remodeling [29,30]. W, on the other hand, improves the mechanical properties of the scaffolds [31] and promotes bone cell growth due to its bioactivity [32,33]. Bioglass, known for its superior bioactivity [34], enhances the osteoconductivity of the scaffold, promoting faster bone growth and stronger integration with host tissues. It uniquely forms a hydroxycarbonate apatite (HCA) layer on its surface upon contact with body fluids, mimicking the mineral phase of bone. This HCA layer facilitates a strong bond between the scaffold and the surrounding bone tissue, significantly enhancing biointegration [29,30]. It also plays a crucial role in stimulating cellular activity and the release of ions that are beneficial for bone regeneration.

The aim of this study was to manufacture scaffolds from PLA/PCL/PPF/PEG-based polymer blends mixed with inorganic fillers, i.e., TCP, W, and BG. Adding these ceramic particles to polymer blends is expected to increase the mechanical strength of the scaffolds, modify degradation profiles, improve their bioactivity, and finally accelerate the regeneration rate of bone tissue. To the best of our knowledge, composite scaffolds with such particular microstructures and compositions have not been reported in the literature. Thus, it is of key importance to characterize their structure and physicochemical properties, as well as biological performance on model cells. We expect that such composite scaffolds may become effective materials in BTE.

## 2. Results

### 2.1. Morphology and Microstructure of the Fabricated Scaffolds

**Figure 1:**
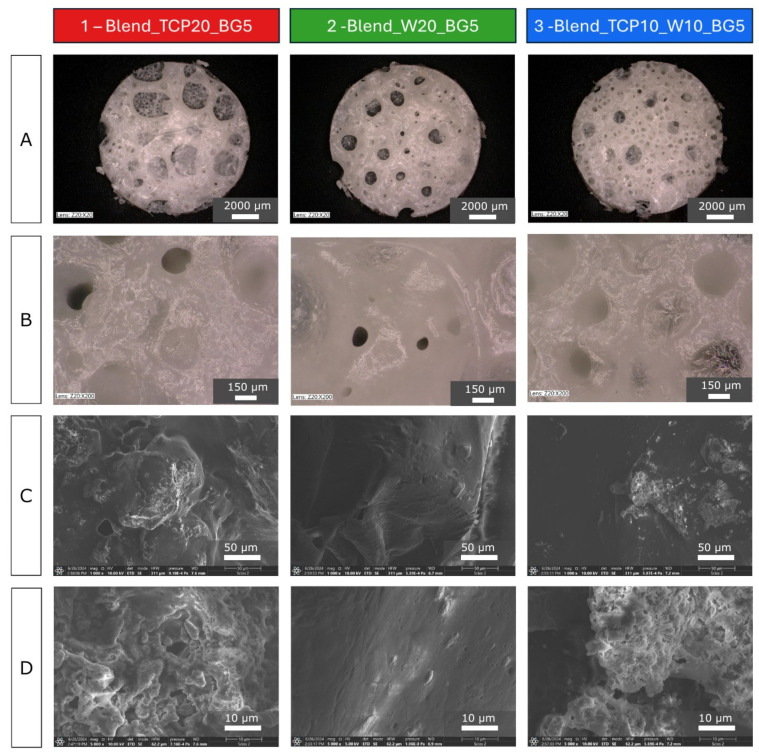
Gross morphology (**A**), optical microphotographs (**B**), and SEM microphotographs (**C**,**D**) of manufactured composite scaffolds.

Optical microscope images (Figure 1A) show the porous structure of all of the samples. At higher magnification (Figure 1B), numerous smaller pores were visible in addition to the large open ones. The pore sizes varied between 50 and 1600 µm. This type of hierarchical microstructure effectively replicates the structure of spongy bone. The surface of samples 1 (Blend_TCP20_BG5) and 3 (Blend_TCP10_W10_BG5) appears rather rough, probably due to the addition of TCP particles, while sample 2 (Blend_W20_BG5), where there is no TCP addition, is smooth. To better understand the microstructure, scanning electron microscopy (SEM) images were taken at different magnifications (Figure 1C,D). SEM observations confirmed previous speculations about the surface of the samples. On the samples with TCP (1 and 3) a higher number of bulges are visible, compared to sample 2, which is rather smooth. Some particles covered with a polymer layer can be observed on the surface of sample 2 (Blend_W20_BG5); however, samples 1 and 3 exhibit a more developed surface area. It seems that the surface topography and microstructure are substantially altered due to the addition of TCP.

### 2.2. FTIR-ATR Spectroscopy 

**Figure 2:**
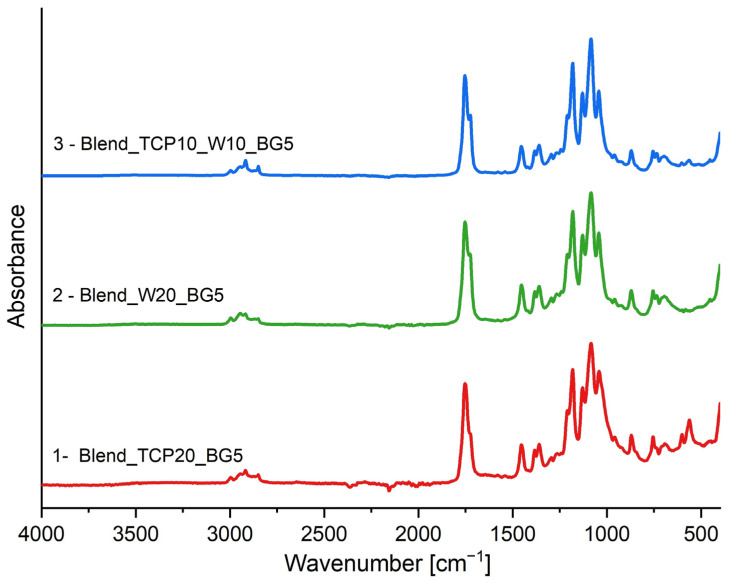
FTIR spectra of manufactured composite scaffolds.

FTIR-ATR spectroscopy analysis was conducted to identify the chemical bonds present in the fabricated scaffolds. Spectra of all of the samples are presented in Figure 2. The double peak that is visible on each spectrum at around 1750 cm^−1^ originates from C=O stretching vibrations and confirms the presence of PLA and PCL in the investigated scaffolds [35,36,37]. The characteristic peaks in the range of 3000–2800 cm^−1^ are generated by the C-H stretching vibrations of the CH_3_ and CH_2_ groups from the polymers present in the blends [35,37]. Moreover, the bands in the range of 1500–1250 cm^−1^ originate from bending vibrations of the C-H groups. Symmetric and antisymmetric bending vibrations of the CH_3_ groups result in bands in the range of 1450–1370 cm^−1^ [35,37]. The band visible at around 1050 cm^−1^ corresponds to υ_3_ phosphate ion (PO_4_^3−^) asymmetric stretching vibrations, confirming the presence of β-TCP in the investigated blends [38,39]. Moreover, the bands at around 600 cm^−1^, visible in the spectra of sample 1 (Blend_TCP20_BG5) and 3 (Blend_TCP10_W10_BG5) with higher intensity for sample 1, correspond to the O-P-O bending in β-TCP [39,40,41]. The characteristic bands around 1100 cm^−1^ and 800 cm^−1^ in each spectrum originate from Si-O-Si stretching and Si-O bending vibrations, respectively [42,43,44]. These vibrations originate from both bioglass and wollastonite; however, the intensity of these peaks is slightly higher in the samples containing W. This suggests that both fillers are present in the investigated samples. All samples have a similar composition, and each spectrum has peaks at similar wavenumbers and with similar intensity, indicating that no new chemical bonds are being created in the fabricated scaffolds.

### 2.3. Differential Scanning Calorimetry 

**Figure 3:**
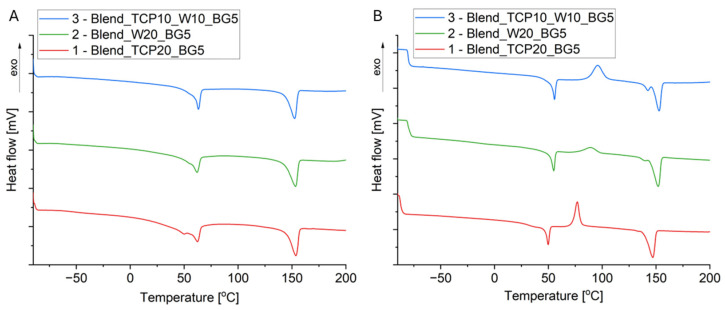
DSC curves of composite scaffolds evaluated after the first (**A**) and second (**B**) heating run.

The manufactured samples were evaluated in terms of their thermal properties. To do so, the DSC analysis was performed in three cycles: heating, cooling, and heating. The DSC curves are presented in Figure 3. To determine the degree of crystallinity (X_c_) of PLA, the following formula was used (1):

(1) $$X_{c}=\frac{{∆H}_{m}}{(1-x_{a})\cdot H_{m}^{0}}\cdot 100\%$$

where

$∆H_{m}$—melting enthalpy of PLA;
$x_{a}$—total mass fraction of the additives, PPF, PCL, and PEG;
$∆H_{m}^{0}$—melting enthalpy of 100% crystalline PLA.

The DSC curves (Figure 3) show the effect of the ceramic additives on the transformations that occur in the scaffolds produced. By comparing the curves, it can be seen that in the first heating run the melting temperature of PLA decreases for samples containing W in their composition compared to the sample with TCP alone; however, this temperature in the second heating cycle does not change significantly for all three samples (Table 1). Cold crystallization is visible for PLA in the second heating run and is in the range of 83–96 °C for all three samples. It can be observed that the cold crystallization temperature increases after the incorporation of W, but the highest value was found for sample 3 (Blend_TCP10_W10_BG5). This suggests that W and W with TCP hinder the PLA cold crystallization process. The glass transition of PCL and PLA in sample 1 (Blend_TCP20_BG5) in the first heating cycle can be observed at temperatures equal to −51 and 48 °C, respectively; however, the temperature for PLA is shifted towards a lower temperature in the second heating cycle. This effect can be attributed to the lower degree of crystallinity, as was found for the second heating run. The effect of the degree of crystallinity on the glass transition temperature of semi-crystalline polymers was investigated by Askadskii et al. [45]. They suggested the influence of the crystallization process and the degree of crystallinity on the mobility of macromolecules within the amorphous domains. It has been revealed that for a low degree of crystallinity, the T_g_ of the amorphous phase increases slowly, whereas for a higher degree of crystallinity, the increase in T_g_ is faster. For PCL, a higher T_g_ was observed as compared to the literature data (−60 °C, [46]), which suggests a higher degree of crystallinity of PCL in the obtained blends. However, an overlap of the thermal effects in the temperature range of 45–60 °C, connected to the glass transition and relaxation of PLA and melting of PCL, makes it difficult to precisely determine the degree of crystallinity of PCL. In addition, the presence of W causes the melting temperature of PLA in the second heating to be maintained at a level similar to that in the first one, and even its slight shift to the right, i.e., to higher temperatures, is observed. The degree of PLA crystallinity depends on the heating cycle and decreases during the second heating. The largest decrease in crystallinity occurs for sample 1 (Blend_TCP20_BG5), and the smallest for sample 2 (Blend_W20_BG5). Sample 3 (Blend_TCP10_W10_BG5) shows a decrease in crystallinity, suggesting that the presence of TCP affects the structure of the polymers during the second heating cycle.

### 2.4. Thermogravimetry

**Figure 4:**
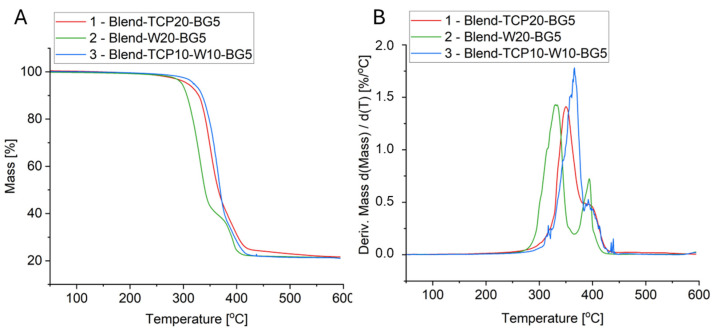
TG (**A**) and DTG (**B**) curves of manufactured composite scaffolds.

The thermal properties and stability of the manufactured scaffolds have been studied using thermogravimetry. Samples were subjected to heating from 40 to 600 °C at a rate of 10 K/min. All three samples show a similar track of TG curves, but depending on the composition, some differences can be observed (Figure 4A). Each sample is characterized by a mass loss of approximately 79% and a residual mass at 600 °C of about 21–22%, which corresponds well to the initial mass of the inorganic fillers in the composition of the samples (Table 2). The thermal stability of the sample with the addition of W is slightly lower than that of the TCP-containing sample; however, interestingly, the addition of both TCP and W increases the thermal stability compared to the samples with only TCP or only W.

Thermal decomposition of PCL, PLA, and PEG for sample 1 (Blend_TCP20_BG5) occurred at temperatures of about 350 °C for PCL, 380 °C for PLA and 390 °C for PEG, which corresponds to the literature findings [35,47,48]. The presence of only W as an inorganic additive in sample 2 (Blend_W20_BG5) causes a significant decrease in the degradation temperatures of PCL and PLA. In the case of the presence of both TCP and W in the composites in sample 3 (Blend_TCP10_W_10_BG5), the changes in degradation temperatures are smaller, meaning that wollastonite is the component that causes these changes. The degradation temperature of PEG in each of the three samples does not change significantly and is around 390 °C. Moreover, adding to the blends both TCP and W enhances the thermal stability of the scaffolds.

### 2.5. Water Contact Angle Measurements

**Figure 5:**
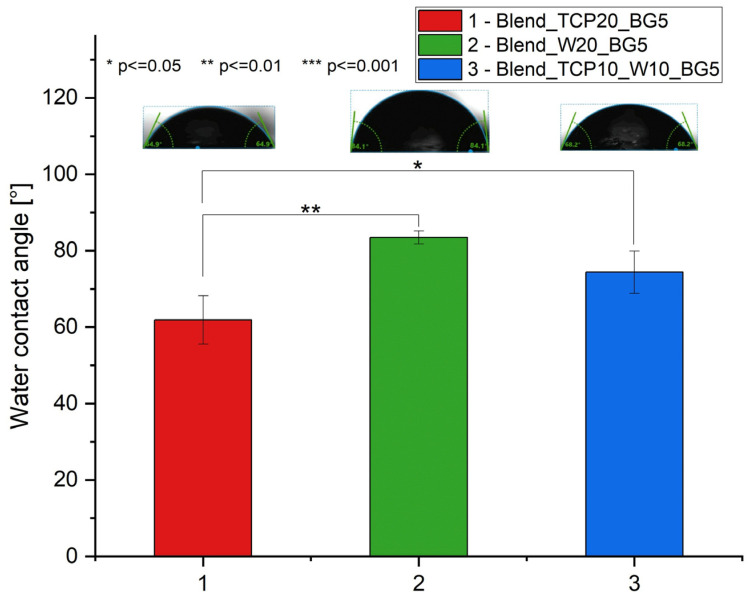
Water contact angle of the manufactured composite scaffolds. Representative droplets are shown above each bar, where *p* * < 0.05, *p* ** < 0.01, *p* *** < 0.001.

To assess the hydrophobic and hydrophilic characteristics of the scaffolds, we evaluated their water contact angles. The measured water contact angles ranged from 61.9 ± 6.3° to 83.4 ± 1.7° (Figure 5), indicating the general hydrophilic nature of the samples. Sample 1 (Blend_TCP20_BG5) exhibited the lowest contact angle, while sample 2 (Blend_W20_BG5) showed the highest, indicating that the addition of TCP enhances hydrophilicity, whereas the addition of W increases hydrophobicity. 

### 2.6. Degradation Study

**Figure 6:**
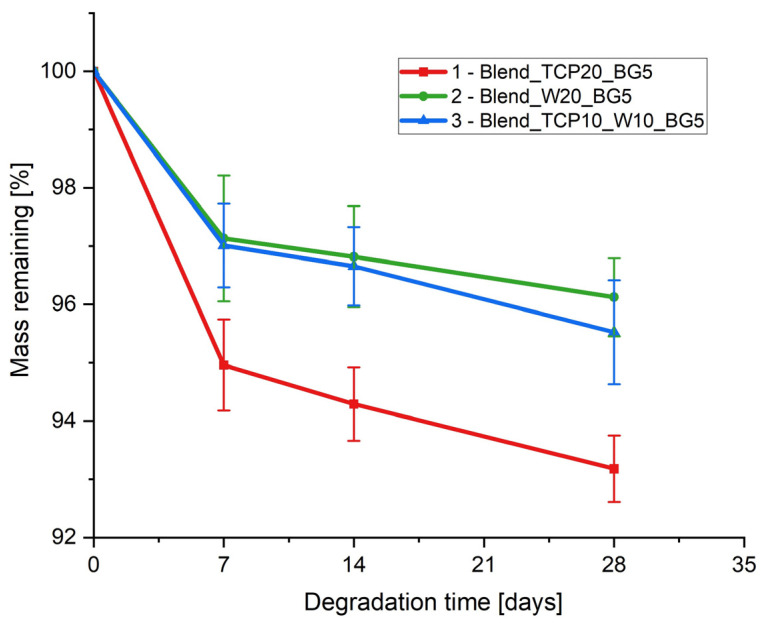
Remaining mass of manufactured composite scaffolds as a function of the incubation time in PBS.

**Figure 7:**
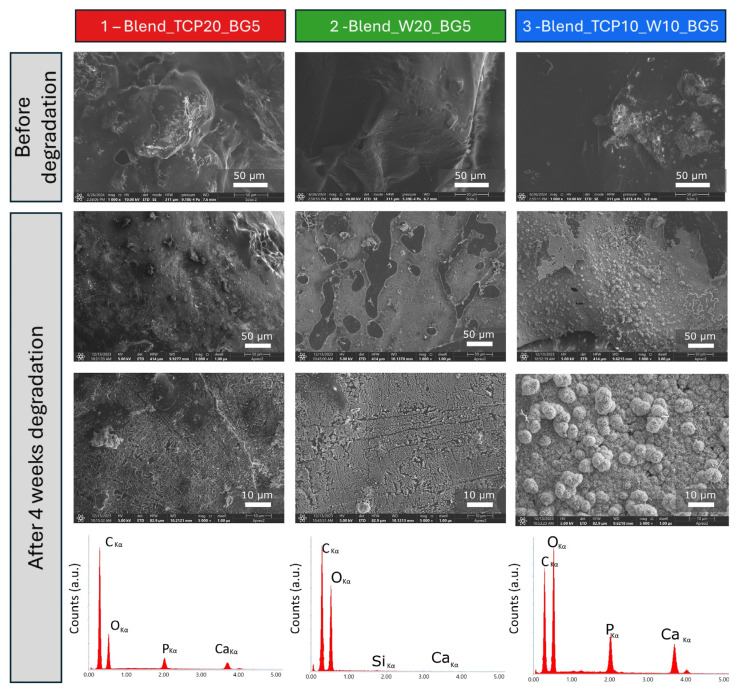
SEM microphotographs of composite scaffolds before (first panel) and after 28 days of degradation study (second and third panels; magnification 1000 and 5000×) and EDS spectra of the samples (fourth panel).

To examine the hydrolytic degradation of the fabricated samples, we subjected them to conditions that simulate human physiological fluids. Specifically, the samples were immersed in PBS and maintained at 37 °C for 28 days. At predetermined time intervals, the sample masses were measured to monitor the degradation rate (Figure 6). Additionally, SEM images were captured before and after the degradation study to assess the morphological changes of the scaffolds (Figure 7). 

The highest mass loss for all samples was observed during the first seven days of the degradation study (Figure 6). Sample 1 (Blend_TCP20_BG5) exhibited the highest degree of degradation, while the degradation for samples 2 (Blend_W20_BG5) and 3 (Blend_TCP10_W10_BG5) was slightly lower with similar changes in mass. These results correspond to the composition of the samples, indicating that adding W to the composite blend can slow down the degradation process of the scaffolds. Sample 1 with only TCP (Blend_TCP20_BG5) exhibited the fastest degradation, while the addition of only W (sample 2 Blend_W20_BG5) caused the slowest one. The presence of both TCP and W (sample 3 Blend_TCP10_W10_BG5) results in the degradation rate between samples 1 and 2. 

SEM microphotographs (Figure 7) present the microstructure of the composite samples before and after degradation in PBS. The results show substantial changes in the microstructure. The surface before degradation seems to be rather smooth with some bulges coming from inorganic filler powders present in the composition covered with the polymeric blend. After 28 days of the degradation study the microstructure appears to be more rough and inorganic components seem to be exposed. Moreover, after 4 weeks of degradation in PBS, the creation of hydroxyapatite-like (HAp) crystals may be observed on the surface of sample 1 (Blend_TCP20_BG5) and sample 3 (Blend_TCP10_W10_BG5). This indicates that the ceramic powders present in the scaffolds, because of their bioactivity, started to react with the PBS environment, creating new calcium phosphate deposits. However, such a phenomenon is not observed on the surface of sample 2 (Blend_W20_BG5), indicating that the creation of HAp-like crystals depends on the presence of TCP in the composition. The largest amount of HAp-like crystals formed on the surface of sample 3 (Blend_TCP10_W10_BG5), indicating the greatest reactivity among the investigated samples. The highest concentration of Ca and P present on the surface of sample 3 is confirmed by the EDS results. Peaks of high intensity for Ca and P are visible for sample 3, while they are lower for sample 1 (Blend_TCP20_BG5) and the lowest for sample 2 (Blend_W20_BG5). That means that sample 2 exhibits the lowest bioreactivity, which is probably related to the absence of TCP in the composition compared to other samples.

### 2.7. Biological Evaluation

**Figure 8:**
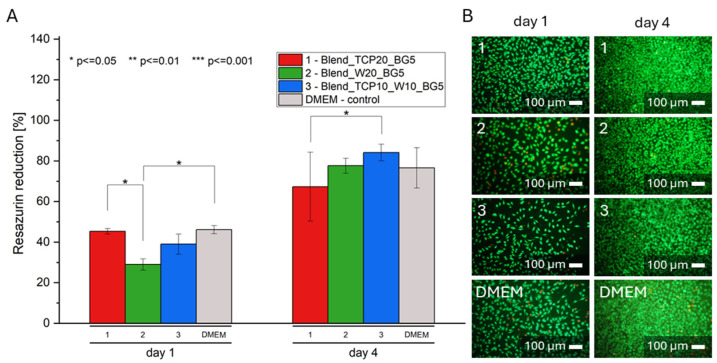
Metabolic activity (**A**) and live/dead staining (**B**) of L929 cells cultured in 10% extracts of composite scaffolds and in control conditions (DMEM), where *p* * < 0.05, *p* ** < 0.01, *p* *** < 0.001.

To investigate the cytotoxicity of the obtained scaffolds, an in vitro study with the use of L929 cells and extracts of the samples was conducted. To do so, cells were seeded in the wells of a 96-well plate (10,000 cells per well) and cultured for 24 h. After that time, the culture medium was replaced with 10% extracts of the scaffolds and the culture continued for 1 and 4 days. The AlamarBlue test (Figure 8A) and live/dead staining (Figure 8B) were performed to study metabolic activity and morphology of the cells tested, respectively. According to the AlamarBlue results, cells were proliferating well in each extract. On day 1, cell viability for the only sample 2 (Blend-W20-BG5) was significantly lower compared to the control conditions. Additionally, on day 4 no differences were observed between the investigated samples and the DMEM control. A similar conclusion can be drawn from the pictures of the living and dead cells. On day 4 much more cells are visible on each sample, compared to day 1. The results indicate that the substances released from the composite scaffolds in the first 24 h are not toxic to the cells used in this study. 

## 3. Discussion

PLA and PCL are popular biodegradable polymers that are widely used in BTE. They are approved by the U.S. Food and Drug Administration (FDA), which makes them candidates of great potential for use in the human body [49,50,51]. Due to their properties, these polymers are characterized by ease of processing [50,52], which results in the possibility of using different methods to process PLA and PCL [50], as well as various forms of polymers, such as membranes [53,54,55], particles [50,56] or scaffolds [13,57,58]. The blend of those two polymers allows one to obtain materials with features that are optimal for BTE. PLA and PCL are being examined as materials used to prepare porous scaffolds with different pore sizes. The variety of pore sizes is crucial for the movement of nutrients and cells as well as waste removal. The smaller the size of the pores, the worse the observed penetration of the cells into the scaffold. Pores that are too small behave as a surface for cells on which they adhere and proliferate rather than penetrating the microstructure [59]. As a result, oxygen and nutrients exchange is altered, leading to cell death within the scaffold [60]. Furthermore, small pores generally result in cell differentiation rather than proliferation, and therefore may not promote osteogenesis due to the creation of a hypoxic environment, which promotes chondrogenesis. Larger pores allow for easier diffusion of body fluids and nutrients, as well as vascularization of newly formed bone tissue [59,60,61]. Furthermore, due to bone ingrowth in pores, porous structures provide better biological fixation, resulting in better biomechanical compatibility [60,62]. On the other hand, even though the presence of microporosity enhances reactivity and ion solubilization and provides interconnected structure and additional areas for protein adsorption, promoting cell adhesion, it reduces the mechanical strength of the scaffold. Moreover, since cell penetration is fundamental for tissue integration and ingrowth, macroporosity plays a crucial role in the regeneration of bone defects [61]. However, increasing the pore size, which promotes new bone growth, affects the mechanical properties of the scaffold. This is why the hierarchical structure of the scaffolds is required to achieve optimal properties [63,64,65]. The gel-casting method is simple and effective in obtaining scaffolds with such an architecture. During the process, random porosity with pore size in the range of 50 to 1600 µm, is being created, meeting the hierarchical structure requirements of the manufactured scaffolds [66]. Interestingly, the cancellous bone is characterized by a pore size in the range of 1–3500 µm; however, the higher porosity, the lower the mechanical properties of the scaffold. It was reported that pore size in the range of 150–200 µm promotes bone formation. The proliferation and differentiation of osteoblasts was presented to be superior in samples with a pore size between 400 and 600 µm. However, samples with larger pores (up to 1100 µm) showed great performance in terms of bone formation in vivo, while smaller ones are reported to enhance vascularization [64,67].

Taking this into account, the obtained hierarchical architecture of the fabricated scaffolds meets the pore size requirements for scaffolds for use in BTE.

Although blending PLA with PCL results in obtaining material with properties better than the polymers themselves, the features of the blend can still be modified. Changing properties of the scaffolds can be made by mixing PLA and PCL with other additives, both organic and inorganic. One of the organic ones can be PEG, which in such composition, modifies the wettability of the samples, as we previously reported [66]. Adding PPF can also allow us to control the properties of the materials, such as the degradation rate [25]. Inorganic fillers, such as bioactive powders, can help to overcome the issues related to the cellular and tissue response [27,33,58,68]. The incorporation of inorganic fillers (TCP, W, BG) into scaffolds is a widely adopted strategy to enhance the properties of the scaffolds. These fillers were chosen because of their specific characteristics that significantly improve the mechanical strength, degradation profiles, and bioactivity of the scaffolds. TCP significantly improves mechanical properties of the scaffolds due to its stiffness [69]. W, similar to TCP, improves the mechanical properties of the scaffolds. The degradation rate of TCP is controllable and matches the rate of new bone formation [70]. This is crucial as it ensures that the scaffold degrades in a manner that supports continuous bone regeneration without premature loss of mechanical integrity. W exhibits a suitable degradation rate that complements the bone healing process. The gradual release of ions during degradation ensures prolonged bioactivity and support for new tissue formation [71]. TCP is highly biocompatible and osteoconductive, which supports the attachment, proliferation, and differentiation of osteoblasts [72]. BG is renowned for its exceptional bioactivity. It forms a HCA layer on its surface when in contact with body fluids, which closely resembles the mineral component of bone [73]. The powders used in this study (TCP, W, BG) were dispersed in the bulk of the polymer blends and were also visible on the samples’ surfaces; however, they seem to be covered with a thin polymer layer in the case of sample 2 (Blend_W20_BG5). A rougher surface can be observed for samples 1 (Blend_TCP20_BG5) and 3 (Blend_TCP10_W10_BG5), and more agglomerates are visible. This may be related to the interaction occurring between TCP and bioglass. According to the literature, replacement of phosphorus in TCP by silicon may occur [74]. Since the surface of sample 2 (Blend_W20_BG5) remained rather smooth, we assume that the differences are due to the presence of TCP. However, all of the samples are characterized by hierarchical architectures with various pore sizes, and the differences can only be observed under higher magnification, i.e., when SEM is used instead of optical microscopy.

According to the FTIR results, the spectra of all of the scaffolds look similar and do not differ remarkably. This is due to the composition of the studied samples. No significant changes are observed in the characteristic bonds of the components present in the blends, as their compositions are similar to each other, resulting in all spectra characterized by bands occurring at similar wavenumbers.

Although the compositions of all of the blends are similar to each other, as confirmed by the FTIR results, different inorganic additives change the thermal properties of the scaffolds. Wollastonite has been reported to enhance the thermal properties of polymers in composites [75]. On the other hand, Canales et al. presented in their study that the presence of bioglass decreases the thermal stability of PLA [76]. The DSC curves show typical transformations that occur in polymers, especially PLA, which is the main component of the blends studied. The peaks from both cold crystallization and melting of PLA in the second heating cycle are slightly shifted to the right (i.e., to higher temperatures) for the samples containing wollastonite in the composition. In addition, the thermal effects for PLA and PCL overlap, so it is not possible to get an accurate interpretation of the transformations occurring in these polymers. The transformation temperatures for PLA for sample 1 (Blend_TCP20_BG5) in the second heating are slightly lowered relative to the first heating, which may be connected to the lower degree of crystallinity; however, this effect seems to be masked by the presence of W in sample 2 (Blend_W20_BG5) and sample 3 (Blend_TCP10_W10_BG5), and the temperatures for these samples remain at similar levels in both cycles. In general, the temperatures in the second heating cycle for all scaffolds are not significantly different from the first one, suggesting that the sample preparation process does not significantly affect thermal transformations occurring in the blends studied. All blended polymers in composite scaffolds start to degrade at temperatures above 300 °C [77,78,79], which correlates with the TG results obtained, where mass decreases are observed in such temperature ranges. All samples are characterized by a residual mass above 21% of the initial mass, which correlates well with the theoretical share of the inorganic additives. In all samples, we can observe decomposition temperatures for PCL, PLA, and PEG, but not for PPF, which may be due to the fact that such effects for all the polymeric blends studied may overlap. The thermal stability is dependent on the sample composition and is the highest for sample 3 (Blend_TCP10_W10_BG5). Interestingly, the thermal stability of sample 2 (Blend_W20_BG5) is slightly lower than that of sample 1 (Blend_TCP20_BG5), suggesting the influence of different inorganic additives on the thermal properties of the tested scaffolds.

Interestingly, the reactivity of different inorganic powders present in the investigated samples can be confirmed by a degradation study. After 4 weeks of incubation in PBS, hydroxyapatite-like crystals are created on the surface of all of the samples, especially sample 3. This is a sign of the bioactivity of all of the inorganic powders used. According to the literature, calcium and phosphate ions are being released during β-TCP degradation, supporting osteoblast activity. Moreover, the bioglass present in the composition of the samples is characterized by its ability to produce a layer of HAp on its surface due to reaction with body fluids, creating an environment promoting bone growth. Interestingly, wollastonite has similar properties and forms a HAp-like layer on its surface when immersed in simulated body fluids [80]. Such properties and behavior of the tested inorganic powders appear to enhance the bioactivity over time, indicating higher bioactivity and biocompatibility of the materials after a certain time of degradation. The degradation rate of the scaffolds depends on their composition [81]. Sample 1 (Blend_TCP20_BG5) exhibits the fastest degradation, while sample 2 (Blend_W20_BG5) exhibits the slowest one. It appears that the higher the concentration of W in the sample, the slower the degradation rate. Interestingly, a similar correlation can be observed for the wettability test. The highest water contact angle was equal to 83.4 ± 1.7° (2 Blend_W20_BG5), while the lowest one was 61.9 ± 6.3° (1 Blend_TCP20_BG5). Samples containing W in the composition show significantly higher hydrophobicity compared to the sample with only TCP. Mixing both TCP and W (3 Blend_TCP10_W10_BG5) results in a water contact angle value between those for samples 1 and 2; however, no statistically significant differences are visible between samples 2 and 3.

Biological evaluation using sample extracts showed that the substances released from the prepared scaffolds during the first 24 h are not toxic to L929 cells. Compared to control conditions, a statistical difference in metabolic activity on the first day of culture occurs only for sample 2 (Blend_W20_BG5); however, on the fourth day of culture, no statistical differences are observed between the samples and the control. Although the cells are metabolically active and proliferate independently of the sample, a slightly better result can be observed for sample 3 (Blend_TCP10_W10_BG5). This can be related to the composition of the investigated samples. Calcium and phosphate ions released from β-TCP, as well as calcium and silicon ions from wollastonite, were reported to contribute to the bioactivity of both TCP and W. These ions are crucial in the bone mineralization process by supporting osteoblast activity. Furthermore, silicon ions support collagen production and the formation of vascular endothelial growth factor (VEGF), thus promoting angiogenesis. Moreover, the resulting HAp layer formed on the surface of the samples enhances bone growth by promoting osteoblast adhesion and proliferation [80,82]. Every material interacts with cells physically and chemically. Chemical interaction depends on the chemical composition, the presence of chemical groups and ions, and other degradation products of the material [83]. The effects of polymer degradation products occurring by hydrolysis or enzymatic digestion depend on the chemical properties of the polymer and are not difficult to determine. However, it is challenging to predict the effects of products released as a result of cellular activity, and more research is still needed to understand the mechanisms underlying these activities [84]. Inorganic ions, such as calcium, phosphorus, and silicon, which are released during material degradation, are involved in bone metabolism, angiogenesis, bone tissue formation, and mineralization. They act as cofactors for enzymes and therefore affect signaling pathways and stimulate metabolic effects that occur during tissue formation [85]. The presence of calcium and phosphate ions affects local pH, which in turn affects the viability of osteoblasts and osteoclasts. In addition, increased concentrations of calcium and phosphate ions can promote bone mineral formation and affect the expression of genes related to osteogenic differentiation of bone cells [86]. Moreover, according to the literature, inorganic phosphate stimulates the expression of Gla protein, a key regulator of bone formation. In addition, Si is known to be an essential element in the metabolic processes involved in bone formation and calcification. Moreover, Si is able to induce hydroxyapatite precipitation [85].

Analysis of the results of the conducted experiments as well as the literature reports show that sample 3 (Blend_TCP10_W10_BG5) seems to have the best properties amongst the other samples tested. It has the best thermal stability. Both surface hydrophilicity and degree of degradation for sample 3 are between sample 1 (Blend_TCP20_BG5) and sample 2 (Blend_W20_BG5), which means that mixing different inorganic additives also allows the properties of the final composites to be controlled. Furthermore, due to degradation, hydroxyapatite-like crystals are formed on the surfaces of the samples, especially sample 3 (Blend_TCP10_W10_BG5), which allows us to state that it exhibits the best bioactive properties. Moreover, sample 3 (Blend_TCP10_W10_BG5) showed the best performance during the initial biological studies with model cells on day 1, although on day 4 of culture no statistical differences were observed for any sample compared to the control.

The study showed that by changing the weight ratio of TCP to W in the polymer blends, we can control not only the thermal properties of the scaffolds but also the surface wettability and degradation kinetics. Importantly, these changes have no negative impact on biological performance, as studied on L929 cells. Although the composites appear to meet the requirements for use in bone tissue regeneration, further research is needed to fully optimize and control the properties of the materials manufactured.

## 4. Materials and Methods

### 4.1. Materials

Poly(lactic acid) (PLA, Ingeo Biopolymer 3052 D, Mn = 180,000 Da) was obtained from Nature Works LLC, courtesy of Natur Tec Pvt Ltd., Chennai, Tamil Nadu, India. Poly(propylene fumarate) was prepared according to Kasper et al. [87]. Poly(ε caprolactone) (PCL, Mn = 80,000 Da), poly(ethylene glycol) (PEG, Mn = 600 Da), and bioactive glass powder (BG, 45S, particle size of 0.2–500 µm) were purchased from Sigma-Aldrich, Steinheim, Germany. β-tricalcium phosphate (TCP, analytical reagent grade, particle size < 500 µm) was provided by Sisco Research Laboratories Pvt. Ltd. (Sisco Labs, Mumbai, India). Wollastonite (W) was purchased from Otto Chemie Pvt. Ltd., Mumbai, India. Dichloromethane and chloroform were purchased from Merck KGaA, Darmstadt, Germany. Phosphate buffered saline (PBS) was obtained from VWR Life Science, Radnor, PA, USA. The L929 mouse fibroblast cell line was provided by the American Type Culture Collections (Manassas, VA, USA). Dulbecco’s modified Eagle medium (DMEM), fetal bovine serum (FBS), penicillin and streptomycin mixture, amino acids, and sodium pyruvate for cell culture were purchased from PANBiotech, Aidenbach, Germany. Calcein AM, propidium iodide, and resazurin were provided by Sigma-Aldrich, Steinheim, Germany.

### 4.2. Scaffolds Manufacturing

To prepare polymer scaffolds, a gel-casting method combined with rapid heating was used, as we described previously [66]. Different compositions of the polymers and inorganic fillers were prepared, according to Table 3. The components were mixed and dissolved in DCM, while stirring at a speed of 300 rpm. Then, the obtained solution was poured onto a glass plate preheated to 70 °C. The evaporation process resulted in the creation of pores of various sizes. To reduce the brittleness of PLA, it was blended with PCL and used as a base for all samples. PEG acted as a plasticizer, while PPF was intended to support infiltrating cells and tissues in the body. Moreover, the addition of the inorganic fillers, such as *β*-TCP and W, as well as bioglass powder, was expected to improve bioactivity and mechanical properties of the scaffolds. Different amounts of TCP and W (0, 10 or 20% of each) were added to the polymer blends, while the amount of BG was kept constant at the level of 5%.

### 4.3. Optical and Scanning Electron Microscopy

An optical microscope (VHX-900F, Keyence, Mechelen, Belgium) and a scanning electron microscope with energy dispersive spectrometer (SEM-EDS, Aero S, Thermo Fisher Scientific, Waltham, MA, USA) were used to observe the microstructure and morphology of the scaffolds obtained. For SEM, the samples were glued on holders and sputter coated with a thin carbon layer and then observed at magnifications of 1000, 5000 and 10,000×.

### 4.4. Fourier Transform Infrared Spectroscopy

To evaluate the composition of the manufactured scaffolds, the attenuated total reflectance-Fourier transform infrared spectroscopy (ATR-FTIR, Tensor 27, Bruker, Billerica, MA, USA) in the wavenumber range of 4000 to 500 cm^−1^ was used. Samples were placed on the diamond crystal and spectra were recorded by averaging 16 scans at a resolution of 1 cm^−1^.

### 4.5. Differential Scanning Calorimetry

The thermal characteristics of the prepared scaffolds were examined using differential scanning calorimetry (DSC, Mettler Toledo DSC1 calorimeter, Greifensee, Switzerland). The samples were placed in aluminum pierced pans and the test was performed in a nitrogen atmosphere (flow 30 mL/min). Heating/cooling/heating cycles in a temperature range of −90 °C to 210 °C at a heating/cooling rate of 10 K/min were applied.

### 4.6. Thermogravimetry

Thermogravimetric analysis (TGA, TG 550 Discovery, TA Instruments, New Castle, DE, USA) was used to determine the actual polymer content in the obtained composite scaffolds. Samples were heated at a rate of 10 K/min from 40 °C to 600 °C in open platinum pans under a nitrogen atmosphere (30 mL/min).

### 4.7. Contact Angle

The wettability of the fabricated scaffolds was investigated by measuring the water contact angle. With the use of a drop shape analyzer (DSA 10, KRÜSS GmbH, Hamburg, Germany), a 2 µL drop of deionized water was placed on the surface of the materials at room temperature. Results are given as average values ± standard deviation (SD) of 10 droplets for each sample.

### 4.8. Degradation Rate

The influence of the presence of the inorganic fillers on the degradation rate of the scaffolds was tested. Samples were immersed in PBS (1 g of samples per 100 mL of PBS) and incubated at 37 °C for 4 weeks. After 1, 2, 3, and 4 weeks, samples were removed from the solution, rinsed with MilliQ water, freeze dried, and weighed. Weight loss (WL) was calculated according to the following Formula (2):

(2) $$WL=\frac{\left(M_{0}-M_{i}\right)}{M_{0}}\cdot 100\;[\%]$$

where

*M*_0_—initial mass of the sample;
*M_i_*—mass of the sample after incubation.

### 4.9. Biological Evaluation on Extracts

To investigate the cytotoxicity of the fabricated polymer scaffolds, L929 fibroblasts were cultured in 10% extracts of the samples. To do so, the samples were immersed in DMEM at a ratio of 1:10 (1 g of sample per 10 mL of DMEM) and incubated for 24 h at 37 °C. Cells, in the number of 10,000 per well, were seeded in a 96-well plate in 100 µL of DMEM supplemented with 10% FBS and 1% of penicillin/streptomycin mixture and were cultured at 37 °C in a 5% CO_2_ atmosphere. After 24 h, the medium was exchanged with prepared extracts previously filtered with 0.22 µm syringe filters and the culture was continued for 4 days. After days 1 and 4, the metabolic activity of the cells was evaluated using the AlamarBlue test, and the morphology of the cells was evaluated by live/dead staining. For the metabolic activity assay, 150 µL of 10% AlamarBlue solution was added to each well and incubated for 3 h. Subsequently, 100 µL of the solution was transferred from each well to a black 96-well plate and the fluorescence was measured (λ_ex_ = 544 nm and λ_em_ = 590 nm, FluoStar Omega, BMG Labtech, Ortenberg, Germany). Resazurin reduction was calculated using the following Formula (3):

(3) $$Resazurin\;reduction=\frac{F_{i}-F_{0}}{F_{100}-F_{0}}\cdot 100\;[\%]$$

where

*F_i_*—fluorescence of the sample;
*F*_0_—fluorescence of a non-reduced solution;
*F*_100_—fluorescence of 100% reduced solution.

For live/dead staining, a solution of calcein AM (0.1%) and propidium iodide (0.1%) in PBS was used. The medium was removed from the wells and replaced with the prepared solution. The plate was then incubated for 20 min in darkness and pictures of the live and dead cells were taken with the use of a fluorescent microscope (ZEISS Axiovert 40 CFL with metal halide illuminator, Oberkochen, Germany).

### 4.10. Statistical Analysis

Results are presented as average values ± standard deviation (SD) of three independent measurements. The Shapiro–Wilk test was used to verify the normal distribution. Statistical significance was determined using a one-way analysis of variance (ANOVA) test with the Tukey’s post hoc test. To do so, Origin 2022 software was used and statistical significance was considered at a probability value lower than 0.05 (* *p* < 0.05, ** *p* < 0.01, *** *p* < 0.001).

## 5. Conclusions

Due to the very complex structure of bone, it is very challenging to manufacture a biomaterial with properties that are optimal for applications in BTE. In our study, we presented a method for obtaining a polymer blend with a hierarchical, porous structure. PLA was modified by blending with other polymers, i.e., PCL, PPF, and PEG, to achieve features more optimal for BTE compared to those of pure PLA. In addition, ceramic powders were embedded in such blends to improve the bioactivity of the obtained materials. The inorganic additives used were characterized by bioactivity, as confirmed by the results of the degradation study carried out in PBS. During the latter experiment, the formation of apatite phases on the materials surface was observed, especially on sample 3 (Blend_TCP10_W10_BG5). In addition to improving bioactivity, the presence of inorganic fillers affected the wettability and thermal properties of the blends, as well as the degradation rate. However, regardless of the composition, the extracts of all of the samples appeared to be non-toxic to L929 cells. Sample 3 (Blend_TCP10_W10_BG5) seems to exhibit the best properties for materials for bone tissue regeneration applications. However, further mechanical, biological, and microbiological studies are required to fully characterize the proposed biomaterials. The described method allows us to obtain a porous material with varying pore sizes, whose properties can be controlled by changing the mass ratios of the components used in the manufacturing process.

## Figures and Tables

**Table 1:** Temperatures of transformations occurring during heating of composite materials: glass transition (T_g_), cold crystallization (T_cc_), melting (T_m_), and degree of crystallinity (X_c_) of PLA.

Sample	T_g_ [°C]		T_cc_ [°C]		T_m_ [°C]		X_c_ [%]	
	1st Heating	2nd Heating	1st Heating	2nd Heating	1st Heating	2nd Heating	1st Heating	2nd Heating
1 (Blend_TCP20_BG5)	−51 (PCL) 48 (PLA)	39 (PLA)	^nd^	83 (PLA)	62 (PCL) 153 (PLA)	53 (PCL) 145 (PLA)	43	35
2 (Blend_W20_BG5)	^nd^	^nd^	^nd^	89 (PLA)	142 (PLA)	144 (PLA)	41	39
3 (Blend_TCP10_W10_BG5)	^nd^	^nd^	^nd^	96 (PLA)	143 (PLA)	146 (PLA)	37	34

^nd^—not determined.

**Table 2:** TGA results for fabricated scaffolds.

Sample	Mass Loss [%]	Char Residue at 600 °C [%]	T_1%_ [°C]	T_3%_ [°C]	T_5%_ [°C]	T_10%_ [°C]	T_50%_ [°C]	T_DTGmax_ [°C]
1 (Blend_TCP20_BG5)	78	22	230	287	308	329	366	351 (PCL) 383 (PLA) 392 (PEG)
2 (Blend_W20_BG5)	79	21	226	287	297	307	341	315 (PCL) 333 (PLA) 394 (PEG)
3 (Blend_TCP10_W10_BG5)	79	21	254	308	318	335	369	324 (PEG) 366 (PLA) 391 (PEG)

**Table 3:** Composition of the scaffolds.

Sample	Component [wt. %]						
	PLA	PCL	PEG	PPF	TCP	W	BG
1—Blend_TCP20_BG5	55	12	5	3	20	-	5
2—Blend_W20_BG5	55	12	5	3	-	20	5
3—Blend_TCP10_W10_BG5	55	12	5	3	10	10	5

## Data Availability

Data will be provided upon request.

## References

[1] Xue N., Ding X., Huang R., Jiang R., Huang H., Pan X., Min W., Chen J., Duan J.-A., Liu P. (2022). Bone Tissue Engineering in the Treatment of Bone Defects. Pharmaceuticals.

[2] Nauth A., Schemitsch E., Norris B., Nollin Z., Watson J.T. (2018). Critical-Size Bone Defects: Is There a Consensus for Diagnosis and Treatment?. J. Orthop. Trauma.

[3] Perez J.R., Kouroupis D., Li D.J., Best T.M., Kaplan L., Correa D. (2018). Tissue Engineering and Cell-Based Therapies for Fractures and Bone Defects. Front. Bioeng. Biotechnol..

[4] Qu H. (2020). Additive Manufacturing for Bone Tissue Engineering Scaffolds. Mater. Today Commun.S.

[5] Koons G.L., Diba M., Mikos A.G. (2020). Materials Design for Bone-Tissue Engineering. Nat. Rev. Mater..

[6] Castañeda-Rodríguez S., González-Torres M., Ribas-Aparicio R.M., Del Prado-Audelo M.L., Leyva-Gómez G., Gürer E.S., Sharifi-Rad J. (2023). Recent Advances in Modified Poly (Lactic Acid) as Tissue Engineering Materials. J. Biol. Eng..

[7] Prasad A. (2021). State of Art Review on Bioabsorbable Polymeric Scaffolds for Bone Tissue Engineering. Mater. Today Proc..

[8] Salvatore L., Gallo N., Natali M.L., Terzi A., Sannino A., Madaghiele M. (2021). Mimicking the Hierarchical Organization of Natural Collagen: Toward the Development of Ideal Scaffolding Material for Tissue Regeneration. Front. Bioeng. Biotechnol..

[9] Liu S., Zheng Y., Hu J., Wu Z., Chen H. (2020). Fabrication and Characterization of Polylactic Acid/Polycaprolactone Composite Macroporous Micro-Nanofiber Scaffolds by Phase Separation. New J. Chem..

[10] Kozaniti F.K., Deligianni D.D., Georgiou M.D., Portan D.V. (2021). The Role of Substrate Topography and Stiffness on MSC Cells Functions: Key Material Properties for Biomimetic Bone Tissue Engineering. Biomimetics.

[11] Chong W.J., Shen S., Li Y., Trinchi A., Pejak Simunec D., Kyratzis I.L., Sola A., Wen C. (2023). Biodegradable PLA-ZnO Nanocomposite Biomaterials with Antibacterial Properties, Tissue Engineering Viability, and Enhanced Biocompatibility. Smart Mater. Manuf..

[12] Ye G., Gu T., Chen B., Bi H., Hu Y. (2023). Mechanical, Thermal Properties and Shape Memory Behaviors of PLA/PCL/PLA-g-GMA Blends. Polym. Eng. Sci.

[13] Shahverdi M., Seifi S., Akbari A., Mohammadi K., Shamloo A., Movahhedy M.R. (2022). Melt Electrowriting of PLA, PCL, and Composite PLA/PCL Scaffolds for Tissue Engineering Application. Sci. Rep..

[14] Ghodrati M., Rafiaei S.M., Tayebi L. (2023). Fabrication and Evaluation of PLA/MgAl2O4 Scaffolds Manufactured through 3D Printing Method. J. Mech. Behav. Biomed. Mater..

[15] Mathieu P., Bascou R., Navarro Oliva F.S., Nesterenko A., Ngo A., Lisiecki I., Guénin E., Bedoui F. (2023). Electrospinning of Ultrafine Non-hydrolyzed Silk Sericin/PEO Fibers on PLA: A Bilayer Scaffold Fabrication. Polym. Eng. Sci.

[16] Elsawy M.A., Kim K.-H., Park J.-W., Deep A. (2017). Hydrolytic Degradation of Polylactic Acid (PLA) and Its Composites. Renew. Sustain. Energy Rev..

[17] Rohner D., Hutmacher D.W., Cheng T.K., Oberholzer M., Hammer B. (2003). In vivo Efficacy of Bone-marrow-coated Polycaprolactone Scaffolds for the Reconstruction of Orbital Defects in the Pig. J. Biomed. Mater. Res..

[18] Serrano M. (2004). In Vitro Biocompatibility Assessment of Poly(ε-Caprolactone) Films Using L929 Mouse Fibroblasts. Biomaterials.

[19] Bartnikowski M., Dargaville T.R., Ivanovski S., Hutmacher D.W. (2019). Degradation Mechanisms of Polycaprolactone in the Context of Chemistry, Geometry and Environment. Prog. Polym. Sci..

[20] Wang J.-Z., You M.-L., Ding Z.-Q., Ye W.-B. (2019). A Review of Emerging Bone Tissue Engineering via PEG Conjugated Biodegradable Amphiphilic Copolymers. Mater. Sci. Eng. C.

[21] Kutikov A.B., Song J. (2015). Biodegradable PEG-Based Amphiphilic Block Copolymers for Tissue Engineering Applications. ACS Biomater. Sci. Eng..

[22] Alge D.L., Bennett J., Treasure T., Voytik-Harbin S., Goebel W.S., Chu T.G. (2012). Poly(Propylene Fumarate) Reinforced Dicalcium Phosphate Dihydrate Cement Composites for Bone Tissue Engineering. J. Biomed. Mater. Res.

[23] Lee K.-W., Wang S., Fox B.C., Ritman E.L., Yaszemski M.J., Lu L. (2007). Poly(Propylene Fumarate) Bone Tissue Engineering Scaffold Fabrication Using Stereolithography: Effects of Resin Formulations and Laser Parameters. Biomacromolecules.

[24] Wang S., Lu L., Yaszemski M.J. (2006). Bone-Tissue-Engineering Material Poly(Propylene Fumarate): Correlation between Molecular Weight, Chain Dimensions, and Physical Properties. Biomacromolecules.

[25] Cai Z., Wan Y., Becker M.L., Long Y.-Z., Dean D. (2019). Poly(Propylene Fumarate)-Based Materials: Synthesis, Functionalization, Properties, Device Fabrication and Biomedical Applications. Biomaterials.

[26] Engebretson B., Sikavitsas V.I. (2012). Long-Term In Vivo Effect of Peg Bone Tissue Engineering Scaffolds. J. Long Term Eff. Med. Implant..

[27] Distler T., Fournier N., Grünewald A., Polley C., Seitz H., Detsch R., Boccaccini A.R. (2020). Polymer-Bioactive Glass Composite Filaments for 3D Scaffold Manufacturing by Fused Deposition Modeling: Fabrication and Characterization. Front. Bioeng. Biotechnol..

[28] Dong J., Uemura T., Shirasaki Y., Tateishi T. (2002). Promotion of Bone Formation Using Highly Pure Porous β-TCP Combined with Bone Marrow-Derived Osteoprogenitor Cells. Biomaterials.

[29] Yamada S. (1997). Osteoclastic Resorption of Calcium Phosphate Ceramics with Different Hydroxyapatite/β-Tricalcium Phosphate Ratios. Biomaterials.

[30] Bohner M., Santoni B.L.G., Döbelin N. (2020). β-Tricalcium Phosphate for Bone Substitution: Synthesis and Properties. Acta Biomater..

[31] Himani J., Purnima J. (2010). Development of Glass Fiber, Wollastonite Reinforced Polypropylene Hybrid Composite: Mechanical Properties and Morphology. Mater. Sci. Eng. A.

[32] Maxim L.D., McConnell E.E. (2005). A Review of the Toxicology and Epidemiology of Wollastonite. Inhal. Toxicol..

[33] Zakaria M.Y., Sulong A.B., Muhamad N., Raza M.R., Ramli M.I. (2019). Incorporation of Wollastonite Bioactive Ceramic with Titanium for Medical Applications: An Overview. Mater. Sci. Eng. C.

[34] Tan F., Naciri M., Al-Rubeai M. (2011). Osteoconductivity and Growth Factor Production by MG63 Osteoblastic Cells on Bioglass-coated Orthopedic Implants. Biotechnol. Bioeng..

[35] Savaris M., Braga G.L., Dos Santos V., Carvalho G.A., Falavigna A., Machado D.C., Viezzer C., Brandalise R.N. (2017). Biocompatibility Assessment of Poly(Lactic Acid) Films after Sterilization with Ethylene Oxide in Histological Study In Vivo with Wistar Rats and Cellular Adhesion of Fibroblasts In Vitro. Int. J. Polym. Sci..

[36] Benkaddour A., Jradi K., Robert S., Daneault C. (2013). Grafting of Polycaprolactone on Oxidized Nanocelluloses by Click Chemistry. Nanomaterials.

[37] Chopra S., Pande K., Puranam P., Deshmukh A.D., Bhone A., Kale R., Galande A., Mehtre B., Tagad J., Tidake S. (2023). Explication of Mechanism Governing Atmospheric Degradation of 3D-Printed Poly(Lactic Acid) (PLA) with Different in-Fill Pattern and Varying in-Fill Density. RSC Adv..

[38] Elhattab K., Bhaduri S.B., Sikder P. (2022). Influence of Fused Deposition Modelling Nozzle Temperature on the Rheology and Mechanical Properties of 3D Printed β-Tricalcium Phosphate (TCP)/Polylactic Acid (PLA) Composite. Polymers.

[39] Saleh A.T., Ling L.S., Hussain R. (2016). Injectable Magnesium-Doped Brushite Cement for Controlled Drug Release Application. J. Mater. Sci..

[40] Afriani F., Dahlan K., Nikmatin S., Zuas O. (2015). Alginate affecting the characteristics of porous β-tcp/alginate composite scaffolds. J. Optoelectron. Biomed. Mater..

[41] Xidaki D., Agrafioti P., Diomatari D., Kaminari A., Tsalavoutas-Psarras E., Alexiou P., Psycharis V., Tsilibary E., Silvestros S., Sagnou M. (2018). Synthesis of Hydroxyapatite, β-Tricalcium Phosphate and Biphasic Calcium Phosphate Particles to Act as Local Delivery Carriers of Curcumin: Loading, Release and In Vitro Studies. Materials.

[42] Herth E., Zeggari R., Rauch J.-Y., Remy-Martin F., Boireau W. (2016). Investigation of Amorphous SiOx Layer on Gold Surface for Surface Plasmon Resonance Measurements. Microelectron. Eng..

[43] Chen W., Liang Y., Hou X., Zhang J., Ding H., Sun S., Cao H. (2018). Mechanical Grinding Preparation and Characterization of TiO2-Coated Wollastonite Composite Pigments. Materials.

[44] Rezaei Y., Moztarzadeh F., Shahabi S., Tahriri M. (2014). Synthesis, Characterization, and In Vitro Bioactivity of Sol-Gel-Derived SiO_2_ –CaO–P_2_ O_5_–MgO-SrO Bioactive Glass. Synth. React. Inorg. M.

[45] Askadskii A., Popova M., Matseevich T., Kurskaya E. (2013). The Influence of the Degree of Crystallinity on the Glass Transition Temperature of Polymers. Adv. Mater. Res..

[46] Sabater I Serra R., Kyritsis A., Escobar Ivirico J.L., Gómez Ribelles J.L., Pissis P., Salmerón-Sánchez M. (2011). Molecular Mobility in Biodegradable Poly(-Caprolactone)/Poly(Hydroxyethyl Acrylate) Networks. Eur. Phys. J. E.

[47] Utomo E., Domínguez-Robles J., Moreno-Castellanos N., Stewart S.A., Picco C.J., Anjani Q.K., Simón J.A., Peñuelas I., Donnelly R.F., Larrañeta E. (2022). Development of Intranasal Implantable Devices for Schizophrenia Treatment. Int. J. Pharm..

[48] Lozano-Sánchez L., Bagudanch I., Sustaita A., Iturbe-Ek J., Elizalde L., Garcia-Romeu M., Elías-Zúñiga A. (2018). Single-Point Incremental Forming of Two Biocompatible Polymers: An Insight into Their Thermal and Structural Properties. Polymers.

[49] Eren Boncu T., Ozdemir N. (2022). Electrospinning of Ampicillin Trihydrate Loaded Electrospun PLA Nanofibers I: Effect of Polymer Concentration and PCL Addition on Its Morphology, Drug Delivery and Mechanical Properties. Int. J. Polym. Mater..

[50] Shekhar N., Mondal A. (2024). Synthesis, Properties, Environmental Degradation, Processing, and Applications of Polylactic Acid (PLA): An Overview. Polym. Bull..

[51] Maity N., Bruchiel-Spanier N., Sharabani-Yosef O., Mandler D., Eliaz N. (2023). Zinc Oxide Nanoparticles Embedded Photo-Crosslinkable PLA-*Block*-PEG toward Effective Antibacterial Coatings. Mater. Adv..

[52] Fernández-Tena A., Pérez-Camargo R.A., Coulembier O., Sangroniz L., Aranburu N., Guerrica-Echevarria G., Liu G., Wang D., Cavallo D., Müller A.J. (2023). Effect of Molecular Weight on the Crystallization and Melt Memory of Poly(ε-Caprolactone) (PCL). Macromolecules.

[53] Ji T., Feng B., Shen J., Zhang M., Hu Y., Jiang A., Zhu D., Chen Y., Ji W., Zhang Z. (2021). An Avascular Niche Created by Axitinib-Loaded PCL/Collagen Nanofibrous Membrane Stabilized Subcutaneous Chondrogenesis of Mesenchymal Stromal Cells. Adv. Sci..

[54] Zhang S., Yan D., Zhao L., Lin J. (2022). Composite Fibrous Membrane Comprising PLA and PCL Fibers for Biomedical Application. Compos. Commun..

[55] More N., Avhad M., Utekar S., More A. (2023). Polylactic Acid (PLA) Membrane—Significance, Synthesis, and Applications: A Review. Polym. Bull..

[56] Iqbal M., Valour J.-P., Fessi H., Elaissari A. (2015). Preparation of Biodegradable PCL Particles via Double Emulsion Evaporation Method Using Ultrasound Technique. Colloid Polym. Sci..

[57] Wang L., Wang C., Zhou L., Bi Z., Shi M., Wang D., Li Q. (2021). Fabrication of a Novel Three-Dimensional Porous PCL/PLA Tissue Engineering Scaffold with High Connectivity for Endothelial Cell Migration. Eur. Polym. J..

[58] Meyhami T., Hassanajili S., Tanideh N., Taheri E. (2024). Three Dimensional Scaffolds of Hybrid PLA/PCL/HA/Silica Nanocomposites for Bone Tissue Engineering. Polym. Bull..

[59] Samourides A., Browning L., Hearnden V., Chen B. (2020). The Effect of Porous Structure on the Cell Proliferation, Tissue Ingrowth and Angiogenic Properties of Poly(Glycerol Sebacate Urethane) Scaffolds. Mater. Sci. Eng. C.

[60] Chen Z., Yan X., Yin S., Liu L., Liu X., Zhao G., Ma W., Qi W., Ren Z., Liao H. (2020). Influence of the Pore Size and Porosity of Selective Laser Melted Ti6Al4V ELI Porous Scaffold on Cell Proliferation, Osteogenesis and Bone Ingrowth. Mater. Sci. Eng. C.

[61] Mohammadi H., Sepantafar M., Muhamad N., Bakar Sulong A. (2021). How Does Scaffold Porosity Conduct Bone Tissue Regeneration?. Adv. Eng. Mater..

[62] Li Y., Yang C., Zhao H., Qu S., Li X., Li Y. (2014). New Developments of Ti-Based Alloys for Biomedical Applications. Materials.

[63] Jodati H., Yılmaz B., Evis Z. (2020). A Review of Bioceramic Porous Scaffolds for Hard Tissue Applications: Effects of Structural Features. Ceram. Int..

[64] Wang C., Xu D., Lin L., Li S., Hou W., He Y., Sheng L., Yi C., Zhang X., Li H. (2021). Large-Pore-Size Ti6Al4V Scaffolds with Different Pore Structures for Vascularized Bone Regeneration. Mater. Sci. Eng. C.

[65] Torres-Sanchez C., Al Mushref F.R.A., Norrito M., Yendall K., Liu Y., Conway P.P. (2017). The Effect of Pore Size and Porosity on Mechanical Properties and Biological Response of Porous Titanium Scaffolds. Mater. Sci. Eng. C.

[66] Pudełko-Prażuch I., Balasubramanian M., Ganesan S.M., Marecik S., Walczak K., Pielichowska K., Chatterjee S., Kandaswamy R., Pamuła E. (2024). Characterization and In Vitro Evaluation of Porous Polymer-Blended Scaffolds Functionalized with Tricalcium Phosphate. J. Funct. Biomater..

[67] Winnett J., Jumbu N., Cox S., Gibbons G., Grover L.M., Warnett J., Williams M.A., Dancer C.E.J., Mallick K.K. (2022). In-Vitro Viability of Bone Scaffolds Fabricated Using the Adaptive Foam Reticulation Technique. Biomater Adv..

[68] Cao C., Huang P., Prasopthum A., Parsons A.J., Ai F., Yang J. (2022). Characterisation of Bone Regeneration in 3D Printed Ductile PCL/PEG/Hydroxyapatite Scaffolds with High Ceramic Microparticle Concentrations. Biomater. Sci..

[69] Di W., Ren H., Li W., Liu D., Sun X. (2024). Fabrication and Characterization of β-TCP/Zn-1Mg Composite Scaffolds for Orthopedic Applications. Mater. Today Commun..

[70] Wang B., Ye X., Chen G., Zhang Y., Zeng Z., Liu C., Tan Z., Jie X. (2024). Fabrication and Properties of PLA/β-TCP Scaffolds Using Liquid Crystal Display (LCD) Photocuring 3D Printing for Bone Tissue Engineering. Front. Bioeng. Biotechnol..

[71] Zenebe C.G. (2022). A Review on the Role of Wollastonite Biomaterial in Bone Tissue Engineering. BioMed Res. Int..

[72] Arpornmaeklong P., Pressler M.J. (2018). Effects of SS-TCP Scaffolds on Neurogenic and Osteogenic Differentiation of Human Embryonic Stem Cells. Anat. Anz..

[73] Hammami I., Graça M.P.F., Gavinho S.R., Jakka S.K., Borges J.P., Silva J.C., Costa L.C. (2024). Exploring the Impact of Copper Oxide Substitution on Structure, Morphology, Bioactivity, and Electrical Properties of 45S5 Bioglass^®^. Biomimetics.

[74] Lopes J.H., Magalhães J.A., Gouveia R.F., Bertran C.A., Motisuke M., Camargo S.E.A., Trichês E.D.S. (2016). Hierarchical Structures of β-TCP/45S5 Bioglass Hybrid Scaffolds Prepared by Gelcasting. J. Mech. Behav. Biomed. Mater..

[75] Wong J.F., Chan J.X., Hassan A.B., Mohamad Z.B., Othman N.B. (2021). Thermal and Flammability Properties of Wollastonite-Filled Thermoplastic Composites: A Review. J. Mater. Sci..

[76] Canales D., Saavedra M., Flores M.T., Bejarano J., Ortiz J.A., Orihuela P., Alfaro A., Pabón E., Palza H., Zapata P.A. (2020). Effect of Bioglass Nanoparticles on the Properties and Bioactivity of Poly(Lactic Acid) Films. J. Biomed. Mater. Res..

[77] Wang S., Lu L., Gruetzmacher J.A., Currier B.L., Yaszemski M.J. (2005). A Biodegradable and Cross-Linkable Multiblock Copolymer Consisting of Poly(Propylene Fumarate) and Poly(ε-Caprolactone): Synthesis, Characterization, and Physical Properties. Macromolecules.

[78] Matumba K.I., Motloung M.P., Ojijo V., Ray S.S., Sadiku E.R. (2023). Investigation of the Effects of Chain Extender on Material Properties of PLA/PCL and PLA/PEG Blends: Comparative Study between Polycaprolactone and Polyethylene Glycol. Polymers.

[79] Ju Z., Brosse N., Hoppe S., Wang Z., Ziegler-Devin I., Zhang H., Shu B. (2024). Thermal and Mechanical Properties of Polyethylene Glycol (PEG)-Modified Lignin/Polylactic Acid (PLA) Biocomposites. Int. J. Biol. Macromol..

[80] Santos G.G.D., Vasconcelos L.Q., Barreto I.C., Miguel F.B., Araújo R.P.C.D. (2022). Wollastonite and Tricalcium Phosphate Composites for Bone Regeneration. Res. Soc. Dev..

[81] Gonçalves Dos Santos G., Borges Miguel I.R.J., De Almeida Barbosa Junior A., Teles Barbosa W., Vieira De Almeida K., García-Carrodeguas R., Lia Fook M., Rodríguez M.A., Borges Miguel F., Correia De Araújo R.P. (2021). Bone Regeneration Using Wollastonite/β-TCP Scaffolds Implants in Critical Bone Defect in Rat Calvaria. Biomed. Phys. Eng. Express.

[82] Murshed M. (2018). Mechanism of Bone Mineralization. Cold Spring Harb. Perspect. Med..

[83] Łukowicz K., Zagrajczuk B., Nowak A., Niedźwiedzki Ł., Laczka M., Cholewa-Kowalska K., Osyczka A.M. (2020). The Role of CaO/SiO2 Ratio and P2O5 Content in Gel-Derived Bioactive Glass-Polymer Composites in the Modulation of Their Bioactivity and Osteoinductivity in Human BMSCs. Mater. Sci. Eng. C.

[84] Saltzman W.M., Kyriakides T.R. (2020). Cell Interactions with Polymers. Principles of Tissue Engineering.

[85] Hoppe A., Güldal N.S., Boccaccini A.R. (2011). A Review of the Biological Response to Ionic Dissolution Products from Bioactive Glasses and Glass-Ceramics. Biomaterials.

[86] Hou X., Zhang L., Zhou Z., Luo X., Wang T., Zhao X., Lu B., Chen F., Zheng L. (2022). Calcium Phosphate-Based Biomaterials for Bone Repair. J. Funct. Biomater..

[87] Kasper F.K., Tanahashi K., Fisher J.P., Mikos A.G. (2009). Synthesis of Poly(Propylene Fumarate). Nat. Protoc..

